# Unplanned Absenteeism: The Role of Workplace and Non-Workplace Stressors

**DOI:** 10.3390/ijerph17176132

**Published:** 2020-08-24

**Authors:** Nur Adibah Mat Saruan, Hanizah Mohd Yusoff, Mohd Fadhli Mohd Fauzi, Sharifa Ezat Wan Puteh, Rosnawati Muhamad Robat

**Affiliations:** 1 Department of Community Health, Faculty of Medicine, Universiti Kebangsaan Malaysia Medical Centre, Jalan Yaacob Latiff, Bandar Tun Razak, Cheras, Kuala Lumpur 56000, Malaysia; adibahsaruan87@gmail.com (N.A.M.S.); fadhli16288@yahoo.com (M.F.M.F.); sh_ezat@ppukm.ukm.edu.my (S.E.W.P.); 2 Ministry of Health Malaysia, Federal Government Administrative Centre, Putrajaya 62590, Malaysia; 3 Occupational and Environmental Health Unit, Selangor State Health Department, No. 1 Wisma Sunway, Jalan Tengku Ampuan Zabedah C 9/C, Seksyen 9, Shah Alam 40100, Malaysia; dr_rosnawati@moh.gov.my

**Keywords:** absenteeism, stress, stressor, nurse, hospital, conflict

## Abstract

Unplanned absenteeism (UA), which includes medically certified leave (MC) or emergency leave (EL), among nurses may disturb the work performance of their team and disrupt the quality of patient care. Currently, there is limited study in Malaysia that examines the role of stressors in determining absenteeism among nurses. Therefore, apart from estimating the prevalence and the reasons of UA among nurses in Malaysia, this study aims to determine its stressor-related determinants. A cross-sectional study was conducted among 697 randomly sampled nurses working in Selangor, Malaysia. Most of them were female (97.3%), married (83.4%), and working in shifts (64.4%) in hospital settings (64.3%). In the past year, the prevalence of ever taking MC and EL were 49.1% and 48.4%, respectively. The mean frequency of MC and EL were 1.80 (SD = 1.593) and 1.92 (SD = 1.272) times, respectively. Meanwhile, the mean duration of MC and EL were 4.24 (SD = 10.355) and 2.39 (SD = 1.966) days, respectively. The most common reason for MC and EL was unspecified fever (39.2%) and child sickness (51.9%), respectively. The stressor-related determinants of durations of MC were inadequate preparation at the workplace (Adj.*b* = −1.065) and conflict with doctors (adjusted regression coefficient (Adj.*b*) = 0.491). On the other hand, the stressor-related determinants of durations of EL were conflict with spouse (Adj.*b* = 0.536), sexual conflict (Adj.*b* = −0.435), no babysitter (Adj.*b* = 0.440), inadequate preparation at workplace (Adj.*b* = 0.257), lack of staff support (Adj.*b* = −0.190) and conflict with doctors (Adj.*b* = −0.112). The stressor-related determinants of the frequency of MC were conflicts over household tasks (Adj.*b* = −0.261), no time with family (Adj.*b* = 0.257), dangerous surroundings (Adj.*b* = 0.734), conflict with close friends (Adj.*b* = −0.467), and death and dying (Adj.*b* = 0.051). In contrast, the stressor-related determinants of frequency of EL were not enough money (Adj.*b* = −0.334), conflicts with spouse (Adj.*b* = 0.383), pressure from relatives (Adj.*b* = 0.207), and inadequate preparation (Adj.*b* = 0.090). In conclusion, apart from the considerably high prevalence of unplanned absenteeism and its varying frequency, duration and reasons, there is no clear distinction in the role between workplace and non-workplace stressors in determining MC or EL among nurses in Malaysia; thus, preventive measures that target both type of stressors are warranted. Future studies should consider longitudinal design and mixed-method approaches using a comprehensive model of absenteeism.

## 1. Introduction

Absenteeism can be a good measure of the health system’s performance and a useful tool in measuring the psychological and physical wellbeing of healthcare workers [1]. It is defined as a failure to attend work according to an established work schedule [2]. Various classifications have been suggested when exploring absenteeism. A few studies categorized it into voluntary and involuntary absenteeism, based on the control ability of the employee [3,4,5]. Other studies have further sub-classified it into the planned and unplanned forms [2]. Planned voluntary absenteeism includes annual leave, study leave, and being off-duty [6]. In contrast, unplanned voluntary absenteeism includes short-term self-certified sickness absence [7], medically certified sickness [8], and others including vehicle breakdown and taking care of a sick child [2,6]. Meanwhile, planned involuntary absenteeism includes absence caused by social obligations such as attending a community meeting [7]. In Malaysia, planned absenteeism is commonly known as annual leave or rest leave, which is typically applied a few days before the intended leave days. In contrast, unplanned absenteeism can be further subdivided into two: (a) sick leave or medically-certified leave (MC) with an accompanying legitimate medical certificate from registered medical practitioners [9] and (b) emergency leave (EL) for any other reasons such as family matters and self-certified sickness [10]. Furthermore, the previous study also has used the term health-related workplace absenteeism to describe the workers’ phenomenon of not attending to work as per working schedule which had been counted by the loss of the number of working hours due to injury or illness [11,12].

Most of the studies conducted abroad to assess sickness absenteeism recorded high prevalence, ranging from 68% to 75% among nurses [5,13,14]. Multiple factors and outcomes of absenteeism among nurses have been identified in previous research. Absenteeism is a side effect of personnel problems, ineffective management, poor working relationships, lack of control over decisions, and overwork [15]. A systematic review found 29 antecedents and nine outcome variables for absenteeism and proposed the Job, Organization, Individual, National and inTerpersonal (JOINT) multilevel conceptual model for investigating absenteeism among nurses. The levels include individual (demographic, personal characteristic, job attitudes, health, and wellbeing), interpersonal (management style and relationship), job (job demand and job control), organization (human resource practices and structure), and national (labor supply and legislation) [1].

In Malaysia, as of 31 December 2017, there were 71,480 and 34,809 nurses working in the public and private sectors, respectively. A total of 106,289 nurses nationwide is equivalent to one nurse to 302 individuals in the population [16]. As for community nurses, 23,771 and 742 community nurses worked in the public and private sectors, respectively. Nurses are described as the main primary workforce in the hospital, as most of the tasks of maintaining continuous support for patients in the wards are performed by nurses [17]. The factors contributing to their absenteeism should, therefore, be taken into account to ensure that healthcare services are well managed. Previous absenteeism studies among nurses working in University hospitals in Malaysia exhibited a higher percentage of EL (65%) compared to MC (52%) [13]. The significant reasons that contributed to both conditions were due to the demand of additional home responsibilities [13]. A study among public service employees in Malaysia also found that stress and personal life problems accounted for up to 69% of the MC [18]. The economic burden often forced them to take up additional jobs and spent more hours working, causing inadequate rest, thus leading to stress [19].

MC and EL can also be an indication of underlying issues of work-related stress [20]. High job demands, organizational injustice and lack of reward are among the job stressors that relate to increased absence due to illnesses [21]. Nevertheless, work stressors specific to the job of nurses are seldom assessed for their associations with unplanned absenteeism. A study on the association of job stress and sickness absence among the general working population in Denmark found that female workers had different strength of association for perceived stress between long-term sickness absence and all-length sickness absence [22]. Thus, to study the association of work stress and unplanned absenteeism among nurses, of whom the majority are female, the duration of MC or EL needs to be analyzed too.

Duclay et al. (2015) found that having less healthcare personnel present at work due to absenteeism would mean that those workers left at work assumed an excessive workload, which caused an imbalance in their health and resulted in a pathological cycle of absenteeism within the institution [23]. A qualitative study found that in addition to the inadequate staffing and workload, absenteeism added pressure during work hours that led to job dissatisfaction [24]. A study among 186 nurses in Limpopo, South Africa also found that absenteeism affected the nurses who remained on duty while their colleagues were absent in the aspect of low morale, psychological stress, and increased workload, consequently jeopardizing patient care with the risk of medical errors [25].

In view of the lack of knowledge on the prevalence and predictors of unplanned absenteeism among nurses, this study was conducted to determine the prevalence (including frequency and duration) of MC and EL among nurses in Malaysia and their reasons for unplanned absenteeism. It aimed to identify potential predictors in terms of workplace and non-workplace stressors, controlling for sociodemographic and occupational profiles. This study is expected to provide initial evidence to health managers to develop strategies that could reduce the number of absent nurses and benefit the organization and the healthcare system.

## 2. Materials and Methods

This study was conducted in the state of Selangor which is the most densely populated state in Malaysia [26]. Selangor has an area of 7950.9 km^2^ with a population density of 819 people per km^2^ [27] with a total population of 5.46 million [26]. The study sample was recruited by simple random sampling. The name list of nurses from all positions working at public hospitals, health clinics (primary healthcare) and district health offices was arranged in one master sheet. Using a prevalence sample size by the Kish formula [28], a reference prevalence of 78% of unplanned absenteeism among nurses in Malaysia [13] was used. Using precision of 3%, the sample size needed was 733 respondents. Next, the respondents were randomly selected using Microsoft Excel (Microsoft, Washington, DC, USA) according to the number of the sample size required.

The inclusion criteria were all Malaysian-nationality registered nurses from the different levels of positions, including matrons, sisters, staff nurses, assistant nurses, midwives/community nurses who have worked at the current workplace for at least six months. Meanwhile, the exclusion criteria were those medically diagnosed with a psychiatric illness or on psychiatric medications for illnesses such as depression, bipolar disorder, anxiety disorder, schizophrenia, and those on long-term sick leave or maternity leave during the study period.

We utilized pencil-and-paper self-reported questionnaires containing sociodemographic (age, gender, marital status, number of children, weight, height, hypertension status and diabetes mellitus status), occupational (workplace setting, work tenure, position, and work schedule), psychological stress (stress status, non-workplace stressor, and workplace stressor), and unplanned absenteeism (frequency, duration, and reason up to the third time taking MC and EL) variables.

MC is operationally defined as self-reported medically certified absenteeism due to medical reasons whereas EL is operationally defined as any other self-reported unplanned absenteeism without prior approval from managers and medical certificate. The frequency and duration of absenteeism were defined as the frequency and the total number of days taking unplanned absenteeism in the past one year for each MC and EL. Reasons for unplanned absenteeism were asked up to the third time of absenteeism (three data points).

Stress status was measured using a validated four-point Likert scale Malay Version of the Personal Stress Inventory: Sign and Symptoms of Stress containing 52 items with 11 subscales. This inventory has been validated in the Malaysian population with a sensitivity of 95.1% and specificity of 77%. The reliability measured by Cronbach alpha was 0.97. The total score of more than 36 indicated that the respondents were having stress [29].

Subsequently, a validated four-point Likert scale Malay Version of the Personal Stress Inventory: Pressures and Demands from Family and Household was used. The inventory contained 12 items which were used to assess the sources of pressure in the non-workplace setting [30]. The inventory consisted of 12 items which included “Not enough money”, “Conflict with spouse”, “Conflicts over household tasks”, “Problems or conflict with children”, “Pressure from relatives or in-laws”, “Fixing up the house”, “Not enough time to spend with family”, “Sexual conflict or frustration”, “Dangerous or stressful surroundings and neighbourhood”, “Conflict with close friend or relatives”, “Personal problem causing strain in family” and “No babysitter”. This questionnaire used a four-point Likert-type scale from “none at all” (0), “a little” (1), “some” (2) and “a great deal” (3). Higher scores indicated higher non-workplace stressors. A total score (ranging from 0 to 36) was obtained by adding the nurse’s responses to all 12 questions. The score above the mean value was categorized as a high score and vice versa. The coverage and relevance of the content were validated by experts in occupational health from academic (university) and service (state health department) side. The reliability using Cronbach alpha was 0.88.

A validated four-points Likert scale Malay Version of Nursing Stress Scale containing 34 items with 7 subscales was used to identify the sources of stress experienced by nurses [30]. It measured the perceived frequency of the occurrence of stress in the nursing environment. The subscales were categorized as; “Workload” (6 items), “Dealing with death and dying” (7 items), “Conflict with doctors” (5 items), “Uncertainty concerning treatment” (5 items), “Lack of staff support” (3 items), “Conflict with other nurses or supervisors” (5 items) and “Inadequate preparation to deal with emotional needs of the patients and their families” (3 items). All items were on potentially stressful situations in the nursing workplace, and the rating was made according to their perceived occurrence. Every item was scored on a four-point Likert-type scale from “never” (0), “occasionally” (1), “frequently” (2) to “very frequently” (3). High scores indicated the more frequent presence of a specific source of stress. A total score ranged from 0 to 102. The score above the mean was categorized as a high score and vice versa. The content was approved by the occupational health experts and the reliability using Cronbach alpha was 0.93.

Data analysis was conducted using SPSS Version 21 (IBM, New York, NY, USA). The incomplete data were dealt with by using multiple imputation techniques whereby the missing data were replaced with the predicted imputed values which correlate with the variables of missing data. This technique was used to ensure the natural variability of the data for valid statistical inference [31]. Statistical analysis began with univariable descriptive analysis, where continuous variables were summarized as mean and standard deviation while categorical variables were presented as frequencies and percentages. Data were further analyzed using simple linear regression, followed by multiple linear regression to identify predictors of frequency and duration of each type of unplanned absenteeism. All potential predictors were initially included, and the elimination was done by the stepwise method. Data were presented as adjusted regression coefficient (Adj.*b*), 95% CI and *p*-value. Significant level was set at *p* < 0.05. Whereas data were collected using dichotomous outcome whether yes or no to determine the predictors between taking MC or not, taking EL or not, taking both MC and EL or not and whether not taking any unplanned leave at all against taking either one leave. The dichotomous outcome was further analyzed using simple logistic regression followed by multiple logistic regression. Data were presented as the adjusted odds ratio (Adj. OR), 95% CI and *p*-value. Significant level was set at *p* < 0.05. This study obtained ethical approval from the Medical Research and Ethics Committee (KKM.NIHSEC.P19-22(6)).

## 3. Results

### 3.1. Descriptive Statistics

The response rate was 95.1% accounts for 697 respondents. Table 1 describes the participants’ sociodemographic profile. The majority of the respondents were female (97.3%) and married (83.4%). Most of them had at least one child (74.8%). Although the majority had no hypertension or diabetes mellitus, more than half of them were overweight/obese.

Table 2 describes the participants’ occupational profile. Most of the respondents worked in a hospital (64.3%) and held positions as staff nurses (61.4%). The majority of them worked in a shift-based work schedule (64.4%) with a mean work tenure of 11.42 (SD = 7.591) years.

Table 3 describes the stressor profiles and stress status. The majority of respondents recorded having no stress (71.88%) with the mean stress score of 25.69 (SD = 20.836). The mean score for non-workplace and workplace stressors was 5.90 (SD = 5.497) and 25.92 (SD = 13.549), respectively.

**Figure 1:**
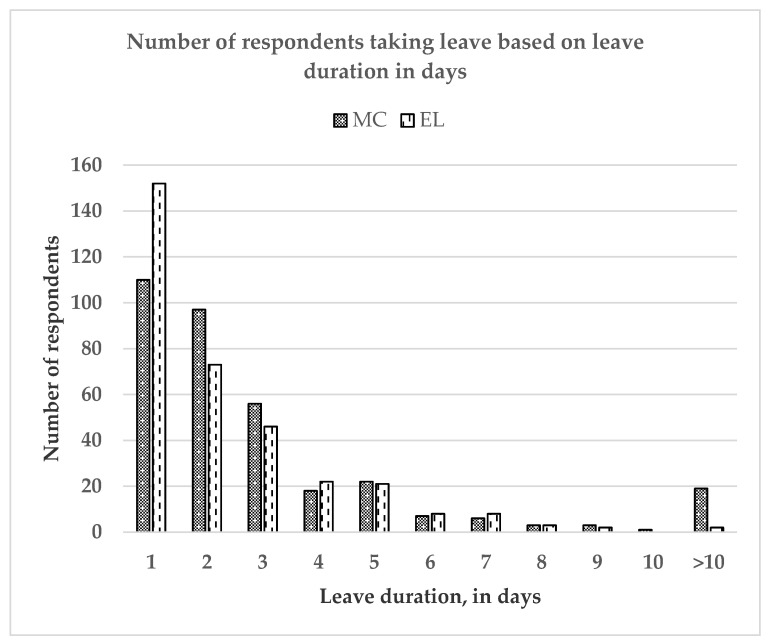
Number of respondents taking leave based on leave duration in days.

**Figure 2:**
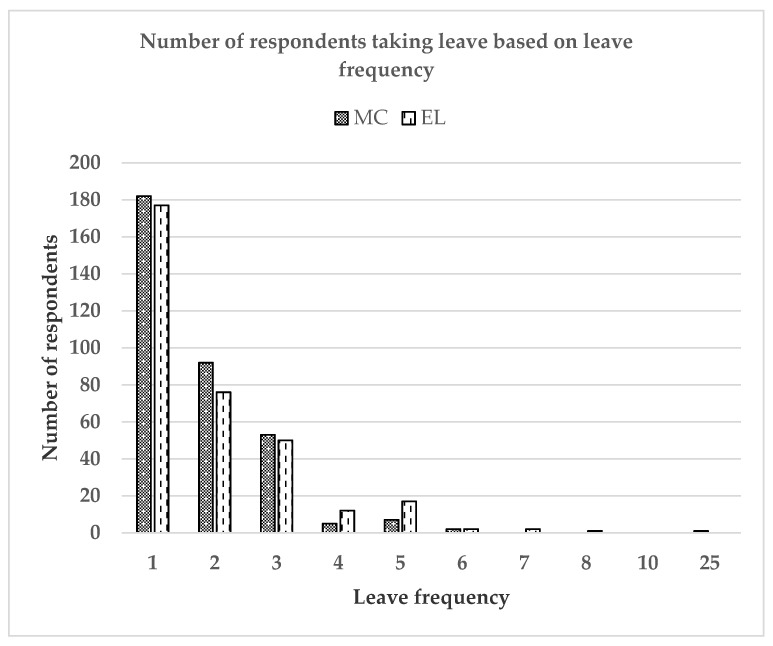
Number of respondents taking leave based on leave frequency.

Table 4 describes the characteristic of MC and EL in term of their prevalence, duration and frequency. The prevalence of ever taking MC and EL in the past one year was 49.07% and 48.35%, respectively. Most respondents took only one-day MC (32.16%) and only once (53.22%). Similarly, most respondents took only one-day EL (45.10%) and only once (52.52%). Subsequently, Figure 1 demonstrates the number of respondents taking leave based on leave duration in days and Figure 2 demonstrates the number of respondents taking leave based on leave frequency.

Table 5 demonstrates the reasons for taking MC and EL. The reasons for MC were mostly medical-related, while the reasons for EL were family-related. The highest reasons for MC were due to unspecified fever (39.18%), non-specified reasons (12.28%), upper respiratory tract infection (URTI)/sinusitis (9.65%) followed by acute gastroenteritis or food poisoning (8.48%) and unspecified dizziness, headache, vertigo, migraine (8.48%). On the other hand, the highest reasons for EL were sick children (51.93%), followed by sick family members (18.10%), and death of family members or relatives (15.73%). Surgery-related MC showed the highest minimum and maximum number of leave days for MC i.e., 10 days and 140 days. Child sickness has been reported as the reason for both MC and EL, which ranges between 5 and 6 days for MC and 1 to 16 days for EL.

### 3.2. Predictors of Those Taking MC, Taking EL, Those Taking MC and EL, and Those neither Take MC nor EL

Table 6 describes the determinants of taking MC, EL, both MC and EL, and neither MC nor EL. Those with older age, and no children, had a higher magnitude of non-workplace stressor related to conflict with close friends and had a lower magnitude of workplace stressor related to inadequate preparation, had higher odds of taking MC. Meanwhile, those who had children and had a higher level of non-workplace stressors related to pressure from relatives had higher odds of taking EL. As for the odds of taking combined MC and EL, the odds are higher among those who ever married, worked in a non-hospital setting, had a lower magnitude of non-workplace stressors related to dangerous surroundings, and had a higher magnitude of workplace stressor related to inadequate preparation.

### 3.3. Predictors of Durations in Days of MC and EL among Those Who Ever Took MC and EL

Table 7 demonstrates the determinants of MC and EL durations among those who ever took MC and EL. The determinants of longer durations of MC were working in a hospital, lower stressors of inadequate preparation and higher stressors of conflict with doctors. In contrast, the determinants of longer durations of EL were having children, being overweight/obese, working in non-shift schedule, higher stressor of conflict with spouse, no babysitter, and inadequate preparation, and lower stressors of sexual conflict, lack of staff support and conflict with doctors.

### 3.4. Predictors of Frequency of MC and EL among Those Who Ever Took MC and EL

Table 8 demonstrates the determinants of MC and EL frequency among those who ever took MC and EL. The determinants of higher frequency of MC were having children, higher magnitude of stressors of no time with family, dangerous surroundings, and death and dying, and lower magnitude of stressors related to conflicts over household tasks and conflict with close friends. On the other hand, the determinants of higher frequency of EL were younger age, having children, being overweight/obese, working in a non-hospital setting, having no stress, a higher level of stressors related to conflicts with spouse, pressure from relatives, and inadequate preparation, and a lower level of stressors related to not enough money.

## 4. Discussions

This study was conducted to determine the prevalence, frequency, duration, and reasons for MC and EL (unplanned absenteeism) and further identify their determinants particularly related to workplace and non-workplace stressors. It was found that almost half of respondents reported ever taking MC or EL which is similar with another study [32]. The mean frequency of MC and EL were two days each, while the mean duration of MC and EL were four and two days, respectively. The top reasons for MC, as the name implied, were mostly medical-related such as unspecified fever, URTI/sinusitis, and acute gastroenteritis (AGE)/food poisoning which is consistent with another study [18]. In contrast, the most common reasons for EL were family-related matters such as child sickness, sick family members, and death of family members. Although most of them were categorised as not having stress (71.9%), both workplace and non-workplace stressors were significantly associated with either MC or EL. These findings signify that MC or EL were not only determined by the direct medical- or family-related reasons mentioned earlier; but stressors may also indirectly play an important role in unplanned absenteeism.

Sociodemographically, it was found that married nurses had higher odds of taking both MC and EL. This finding is similar to a study that showed marriage had a significant effect on absenteeism, as they had to be responsible for other additional family members [33]. On the other hand, nurses with children had higher odds of taking EL, longer duration of EL, and higher frequency of MC. This is consistent with studies that shown that larger family sizes will increase the amount of responsibilities and increase work–family conflict, subsequently resulting in absenteeism [13,34,35]. Apart from that, being overweight/obese had contributed to an increase in frequency and duration of EL. This is supported by one study that reported that overweight increased the risk of absenteeism which may be contributed by the lack of enthusiasm at work [36].

Occupationally, it was found that non-hospital nurses had higher odds of taking both MC and EL. In addition, non-hospital nurses had higher frequency of taking EL but lesser duration of MC. This is consistent with a previous study which reported that those working in the primary care covering clinics had reported a 41% higher incidence of absence during the second year and an increase to 50% in the following year compared to those working in the wards [37]. Our study also found that nurses who worked in a non-shift schedule had higher duration of EL. This finding contradicts with previous studies that showed that the shift schedule had a significant association with absenteeism [38] which could be due to the conflicting responsibilities between working in shifts and attending to family members which could lead to absenteeism [39]. We postulate that this contradictory finding was contributed to by the fact that essential services including child education, banking, and administrative services are provided during office hours, which may influence the decision of nurses who work in a non-shift schedule to take EL to settle their essential non-work-related matters.

Although stress was one of the main culprits of absenteeism which can jeopardise the organisation [40], our study found that stress was associated with lower frequency of EL. We postulate that this could be due to the differential in root causes of stress that indirectly influence absenteeism. For instance, those who experience financial constraints which have been shown to be associated with stress [19] may or may not be absent from work; those who absent may be due to the involvement in part-time job that jeopardize their attendance at work, while those who present may be due to the fear in losing the current job and income. However, this postulation needs to be confirmed in future study using a longitudinal study involving multiple interrelated occupational and non-occupational variables.

Nurses with workplace stressors of inadequate preparation had lower odds of taking MC and had shorter durations of MC but higher duration and frequency of EL. Inadequate preparation in handling work tasks in terms of mental readiness in treating patients tend to make nurses feel irresponsible when managing patients and leave them vulnerable to making mistakes, leaving them no choice but to take the EL. Recent advances in technology and an increasing demand in care requires nurses to develop increasingly higher skill levels that only expose them to more stress than other healthcare professionals [41]. Krohne and Magnussen (2011) stated that those who are equipped with knowledge and preparation for work would promote a good healthy working environment, which prevents withdrawal behaviour [42]. On the other hand, the workplace stressor related to lack of staff support was significantly associated with shorter durations of EL. The lack of managerial support for a work–life balance leads to added pressure for workers to return to work as soon as possible, perhaps even before they are ready to do so. This was in line with a study suggesting that supervisor positive attitudes towards the aspect of the non-work domain will support their employees in handling the competing family demands thus reducing the degree of presenteeism [39].

Conflicts with doctors significantly increased the duration of taking MC but lower duration of EL. Nurses might not have the benefits of taking MC especially during the earlier phases of an illness which result in the nurse being severely ill resulting in a prolonged duration of MC [43]. Accordingly, a study among the Chinese population found that supervisors tend not to believe the reasons given by the workers on sick leave, thus leading to presenteeism which causes further disruption in work productivity [44]. Finally, nurses who were occupationally stressed about death and dying had higher odds of not taking neither MC nor EL. However, if nurses who were stressed about death and dying took MC, they were more likely to have a higher frequency of MC. Facing real-life tragedies left them emotionally disturbed and unable to continue working due to mental illness or disorder [45].

Non-workplace stressors were associated with the frequency or duration of MC and EL in varying directions of influence. For instance, no time with family and dangerous surroundings were associated with a higher frequency of MC but conflict with close friends and conflicts over household tasks were associated with a lower frequency of MC. In addition, pressure from relatives and conflict with spouse were associated with a higher frequency of EL, while conflicts with spouse and no babysitter were associated with a higher duration of EL. Moreover, sexual conflict was associated with a lower duration of EL, but insufficient money was associated with a lower frequency of EL. Similar findings were recorded for workplace stressors which have been discussed earlier. These findings may suggest that the origin of stressors plays an important role in influencing medical- or family-related outcome which consequently determine the aspect of MC and EL. Another possible reason could be the cross-sectional design that is unable to infer causation [46], and it is thus unknown which comes first either the MC/EL or the non-workplace stressors. This could also be due to the interaction between workplace and non-workplace stressors that influence the MC or EL.

In view of the possible interaction of workplace and non-workplace stressors with absenteeism, some working organisations support the introduction of a family-friendly organisational culture by encouraging managers to support family life [47]. Modifying the workplace environment, which is the responsibility of both employees and managers, is necessary given potential for modifiable determinants to control unplanned absenteeism. An absenteeism policy should be in place to ensure that rules are stated clearly, and the daily work process should continue as usual [48]. Flexible working time arrangements can be considered for those who have conflicting responsibilities between work and family and can be applied to those who have illnesses as well. A family-supportive organisational culture at the workplace should be created by getting managers to support the work–life balance. Despite the need to reduce unplanned absenteeism, managers should be concerned for their workers’ general well-being; therefore, MC should be encouraged to those who have acute minor illness or else the upcoming health-related consequences will lead to a worse impact. For instance, those with URTI which could be easily transmitted in a healthcare setting could lead to a longer duration of MC or higher number of workers taking MC if the source workers continue to work despite having the illness.

This study has limitations related to the cross-sectional design that could neither infer causation nor examine the mediating/moderating effect of other variables. Therefore, there is a need to conduct a longitudinal design to examine the interrelationship among workplace and non-workplace stressors, and their causal effects towards absenteeism. The subsequent study should also comprehensively refer to the model of absenteeism to guide researchers on how to tackle the possible determinants acquired from the respondents to explore other factors of unplanned absenteeism. Other than that, future studies can be commenced qualitatively to determine the specific reasons for unplanned absenteeism at a different hospital setting.

Apart from cross sectional design, the other limitation of this study was in the exploration of reasons for leave and days of leave for each reason was only up to the third time of frequency. Therefore, we were unable to capture the reasons for unplanned absenteeism that exceeded more than three times. Furthermore, the findings were self-reported; hence, we could not verify the validity of the number of days, frequency, and reasons for unplanned absenteeism. Other than that, this study had induced recall bias as respondents tended to remember obvious common reasons instead of uncommon ones; thus, the absolute reasons for unplanned absenteeism should be interpreted with caution. Another limitation was a misclassification on the reasons for unplanned absenteeism that might have been wrongly stated by respondents including medical appointments/procedure and surgery. Having advanced notice of an upcoming leave due to these reasons could be classified under planned absenteeism.

## 5. Conclusions

To conclude, the prevalence of MC and EL among nurses working in Malaysia for the past one year was 49% and 48%, respectively. A majority of the subjects took both MC and EL for only once and for only a one-day duration for the past year. The most common reason for MC and EL was unspecified fever and sick children, respectively. There is no clear distinction between workplace and non-workplace stressors for MC, EL or both. Both workplace and non-workplace stressors showed different significance, magnitude and direction of association towards the duration or frequency for MC and EL. Nevertheless, preventive measures should be taken by targeting modifiable factors, which involve getting managers on board and promoting a stress-free environment in the workplace. Future study should consider employing a longitudinal design that combines both qualitative and quantitative method based on a comprehensive model of absenteeism.

## Figures and Tables

**Table 1:** Participants’ sociodemographic profile.

Variables, n = 697	Min.	Max.	n (%)	Mean (SD)
Age, in years	20	59		34.67 (8.148)
Gender				
Male			19 (2.73)	
Female			678 (97.27)	
Marital Status				
Single			100 (14.35)	
Married			581 (83.36)	
Separated/Divorced/Others			16 (2.30)	
No. of Children	0	7		1.84 (1.516)
None			176 (25.25)	
At least one child			521 (74.75)	
Body Mass Index (BMI), in kg/m^2^				25.79 (5.508)
Underweight (<18.50 kg/m^2^)			32 (4.59)	
Normal (18.50 to 24.99 kg/m^2^)			321 (46.05)	
Overweight (25.00 to 29.99 kg/m^2^)			205 (29.41)	
Obese (30.00 kg/m^2^ and above)			139 (19.94)	
Other Comorbid				
Having hypertension			53 (7.60)	
Having diabetes mellitus			34 (4.88)	

**Table 2:** Participants’ occupational profile.

Variables, n = 697	Min.	Max.	n (%)	Mean (SD)
Workplace				
Hospital			448 (64.28)	
Public Health and Primary Healthcare			249 (35.72)	
Work tenure as nurse, in years				11.42 (7.591)
Position				
Community Nurse			162 (23.24)	
Staff Nurse/Midwife			428 (61.41)	
Sister ^a^			90 (12.91)	
Matron ^b^			17 (2.44)	
Work Schedule				
Non-Shift Work			248 (35.58)	
Shift Work			449 (64.42)	

^a^ ‘Sister’: A nurse in charge who is responsible for the immediate functioning of the unit; ^b^ ‘Matron’: chief nurse who in charge of nursing in a hospital and the head of the nursing staff.

**Table 3:** Stressors profile and stress status.

Variables, n = 697	n (%)	Mean (SD)
STRESS STATUS		25.69 (20.836)
Non-stress (Score less than 36)	501 (71.88)	
Stress (Score 36 and above)	196 (28.12)	
NON-WORKPLACE STRESSOR		5.90 (5.497)
Not enough money		0.68 (0.796)
Conflicts with spouse		0.48 (0.693)
Conflicts over household tasks		0.48 (0.682)
Conflicts with children		0.36 (0.598)
Pressure from relatives		0.44 (0.713)
Fixing up of house		0.43 (0.681)
No time with family		1.08 (0.966)
Sexual conflicts		0.21 (0.513)
Dangerous surroundings		0.42 (0.663)
Conflict with close friends		0.40 (0.603)
Personal problems cause strain		0.40 (0.636)
No babysitter		0.51 (0.797)
WORKPLACE STRESSOR		25.92 (13.549)
Workload		8.39 (3.640)
Death and dying		4.39 (3.831)
Inadequate preparation		1.80 (1.572)
Lack of staff support		2.11 (1.908)
Uncertainty concerning treatment		3.19 (2.429)
Conflict with doctors		3.22 (2.552)
Conflict with other nurses		2.81 (2.453)

**Table 4:** Unplanned absenteeism profile.

Variables, n = 697	Medically-Certified Leave (MC)				Emergency Leave (EL)			
	n (%)	Min.	Max.	Mean (SD)	n (%)	Min.	Max.	Mean (SD)
Prevalence								
Never taken	355 (50.93)				360 (51.65)			
Ever taken	342 (49.07)				337 (48.35)			
Duration in days of taking leave ^a^		1	140	4.24 (10.355)		1	16	2.39 (1.966)
Frequency of taking leave ^a^		1	25	1.80 (1.593)		1	8	1.92 (1.272)

^a^ Among those who had ever taken a medically certified leave (n = 342) or emergency leave (n = 337), respectively.

**Table 5:** Reasons for unplanned absenteeism ^a^.

Variables	n (%)	No. of Leave Days for Each Reason			
		Min	Max	Mean (SD)	Total
MEDICALLY-CERTIFIED LEAVE (MC) ^b^					
Unspecified fever	134 (39.18)	1	4	1.54 (0.732)	206
Non-specified	42 (12.28)	1	33	3.17 (5.231)	133
Upper respiratory tract infection, sinusitis	33 (9.65)	1	9	2.00 (1.581)	66
AGE, food poisoning	29 (8.48)	1	4	1.38 (0.820)	40
Dizziness, headache, vertigo, migraine	29 (8.48)	1	4	1.72 (0.751)	50
Unspecified symptoms (ache, cough)	26 (7.60)	1	5	1.73 (1.079)	45
Tooth-related pain and procedure	23 (6.73)	1	5	1.65 (1.027)	38
Eye-related (e.g., conjunctivitis)	18 (5.26)	1	5	2.44 (1.338)	44
Trauma-related (fracture, tissue injury)	16 (4.68)	1	60	8.25 (15.159)	132
Medical appointment/procedure	15 (4.29)	1	4	1.80 (1.014)	27
Others (otitis, pneumonia, burn, allergy)	12 (3.51)	1	10	3.33 (2.964)	40
Surgery (I&D, laparotomy, TAHBSO)	11 (3.22)	10	140	39.45 (38.816)	434
AEBA	10 (2.92)	1	7	2.50 (2.121)	25
CVS-related (ACS, hypertension, stroke)	8 (2.34)	1	30	5.75 (9.867)	46
MSD (CTS, PID, backache)	7 (2.05)	1	16	4.71 (5.499)	33
Viral fever, dengue fever	7 (2.05)	1	7	3.71 (2.498)	26
GERD, gastritis	5 (1.46)	1	3	2.00 (1.000)	10
Urinary tract infection	4 (1.17)	1	7	3.25 (2.630)	13
Menstrual-related	3 (0.88)	1	1	1.00 (0.000)	3
Child sickness	2 (0.58)	5	6	5.50 (0.707)	11
EMERGENCY LEAVE (EL) ^c^					
Child sickness	175 (51.93)	1	16	2.23 (1.789)	390
Sick family members or relatives	61 (18.10)	1	7	2.18 (1.658)	133
Death of family members	53 (15.73)	1	5	1.51 (0.993)	80
Unspecified reasons	35 (10.39)	1	5	1.71 (1.073)	60
Child matters except sickness	31 (9.20)	1	3	1.35 (0.709)	42
Self-certified health problem	21 (6.23)	1	3	1.33 (0.658)	28
Unspecified family- or self-related matters	16 (4.75)	1	3	1.38 (0.719)	22
Vehicle problem or MVA	9 (2.67)	1	1	1.00 (0.000)	9
Medical appointment	1 (0.30)	1	1	1.00 (0.000)	1
Others	1 (0.30)	1	1	1.00 (0.000)	1

^a^ We sampled only the first three unscheduled absenteeism; ^b^ Denominator is the respondents who took MC (n = 342); ^c^ Denominator is the respondents who took EL (n = 337); AGE: acute gastroenteritis; MVA: motor vehicle accident; MSD: musculoskeletal disease; CTS: carpal tunnel syndrome; PID: prolapse intervertebral disc; GERD: gastroesophageal disease; I&D: incision and drainage; TAHBSO: total abdominal hysterectomy and bilateral salpingo-oophorectomy; AEBA: acute exacerbation bronchial asthma; ACS: acute coronary syndrome; CVS: cardiovascular.

**Table 6:** Predictors of taking medically certified leave (MC), emergency leave (EL), both MC and EL, and neither took MC nor EL.

Variables, n = 697	Exp (B) (95% CI) ^a^			
	MC ^b^	EL ^c^	MC + EL ^d^	None ^e^
SOCIODEMOGRAPHIC				
Age	1.029 (1.004, 1.056)			
Marital status (Ref. = ever married)			0.252 (0.135, 0.473)	2.193 (1.404, 3.425)
Having children (Ref. = have children)	2.120 (1.323, 3.395)	0.414 (0.238, 0.718)		
BMI (Ref. = overweight/obese)				
OCCUPATIONAL				
Workplace (Ref. = hospital)			1.696 (1.195, 2.407)	0.625 (0.432, 0.905)
Work schedule (Ref. = non-shift)				
STRESS STATUS (Ref. = yes)				
NON-WORKPLACE STRESSOR				
Not enough money				
Conflicts with spouse				
Conflicts over household tasks				
Conflicts with children				
Pressure from relatives		1.658 (1.228, 2.239)		0.687 (0.516, 0.916)
Fixing up of house				
No time with family				
Sexual conflicts				
Dangerous surrounding			0.712 (0.542, 0.936)	
Conflict with close friends	1.394 (1.007, 1.928)			
Personal problems cause strain				
No babysitter				
WORKPLACE STRESSOR				
Workload				
Death and dying				0.921 (0.875, 0.969)
Inadequate preparation	0.754 (0.644, 0.820)		1.210 (1.082, 1.353)	
Lack of staff support				
Uncertainty concerning treatment				
Conflict with doctors				
Conflict with other nurses				

^a^ Although all variables in the table were included in the adjusted model, only significant results were presented; ^b^ adj.R^2^ = 0.071; ^c^ adj.R^2^ = 0.060; ^d^ adj.R^2^ = 0.083; ^e^ adj.R^2^ = 0.091.

**Table 7:** Predictors of duration in days of MC and EL among those ever took MC and EL.

Variables	Adj.*b* (95% CI) ^a^	
	Duration of MC among Those Ever Took MC (n = 342) ^b^	Duration of EL among Those Ever Took EL (n = 337) ^c^
SOCIODEMOGRAPHIC PROFILE		
Age		
Marital status (0 = never married; 1 = ever married)		
Having children (0 = no children; 1 = have children)		0.781 (0.242, 1.320)
Body mass index (0 = underweight/normal; 1 = overweight/obese)		0.417 (0.019, 0.816)
OCCUPATIONAL PROFILE		
Workplace (0 = non-hospital; 1 = hospital)	3.411 (0.721, 6.101)	
Work schedule (0 = shift; 1 = non-shift)		0.463 (0.039, 0.888)
STRESS STATUS (0 = no; 1 = yes)		
NON-WORKPLACE STRESSOR		
Not enough money		
Conflicts with spouse		0.536 (0.184, 0.888)
Conflicts over household task		
Conflicts with children		
Pressure from relatives		
Fixing up of house		
No time with family		
Sexual conflict		−0.435 (−0.848, −0.022)
Dangerous surrounding		
Conflict with close friends		
Personal problem cause strain		
No babysitter		0.440 (0.166, 0.714)
WORKPLACE STRESSOR		
Workload		
Death and dying		
Inadequate preparation	−1.065 (−1.849, −0.282)	0.257 (0.104, 0.409)
Lack of staff support		−0.190 (−0.322, −0.059)
Uncertainty concerning treatment		
Conflict with doctors	0.491 (0.000, 0.982)	−0.112 (−0.220, −0.003)
Conflict with other nurses		

^a^ Adjusted regression coefficient (all variables in the table were included in this adjusted model; however only significant results were presented); ^b^ Multiple linear regression (Constant = 1.526; adj.R^2^ = 0.027; model assumptions are met); ^c^ Multiple linear regression (Constant = 1.129; adj.R^2^ = 0.132; model assumptions are met).

**Table 8:** Predictors of frequency of MC and EL among those ever took MC and EL.

	Adj.*b* (95% CI) ^a^	
Variables, n = 337	Frequency of MC among Those Ever Took MC (n = 342) ^b^	Frequency of EL among Those Ever Took EL (n = 337) ^c^
SOCIODEMOGRAPHIC PROFILE		
Age		−0.024 (−0.042, −0.006)
Marital status (0 = never married; 1 = ever married)		
Having children (0 = no children; 1 = have children)	0.601 (0.210, 0.991)	0.521 (0.161, 0.881)
OCCUPATIONAL PROFILE		
Body mass index (0 = underweight/normal; 1 = overweight/obese)		0.385 (0.121, 0.648)
Workplace (0 = non-hospital; 1 = hospital)		−0.327 (−0.594, −0.060)
Work schedule (0 = shift; 1 = non-shift)		
STRESS STATUS (0 = no; 1 = yes)		−0.368 (−0.661, −0.076)
NON-WORKPLACE STRESSOR		
Not enough money		−0.334 (−0.523, −0.145)
Conflicts with spouse		0.383 (0.157, 0.610)
Conflicts over household task	−0.261 (−0.519, −0.002)	
Conflicts with children		
Pressure from relatives		0.207 (0.015, 0.398)
Fixing up of house		
No time with family	0.257 (0.066, 0.448)	
Sexual conflict		
Dangerous surrounding	0.734 (0.438, 1.031)	
Conflict with close friends	−0.467 (−0.779, −0.154)	
Personal problem cause strain		
No babysitter		
WORKPLACE STRESSOR		
Workload		
Death and dying	0.051 (0.004, 0.099)	
Inadequate preparation		0.090 (0.006, 0.173)
Lack of staff support		
Uncertainty concerning treatment		
Conflict with doctors		
Conflict with other nurses		

^a^ Adjusted regression coefficient (all variables in the table were included in this adjusted model; however only significant results were presented); ^b^ Multiple linear regression (Constant = 0.912; adj.R^2^ = 0.116; model assumptions are met); ^c^ Multiple linear regression (Constant = 2.077; adj.R^2^ = 0.151; model assumptions are met).
